# Effect of chemical species and temperature on the stability of air nanobubbles

**DOI:** 10.1038/s41598-023-43803-6

**Published:** 2023-10-04

**Authors:** Seyed Mohammad Montazeri, Nicolas Kalogerakis, Georgios Kolliopoulos

**Affiliations:** 1 Department of Mining, Metallurgical, and Materials Engineering, Université Laval, Québec, QC G1V 0A6 Canada; 2 School of Chemical and Environmental Engineering, Technical University of Crete, 73100 Chania, Greece

**Keywords:** Chemical engineering, Environmental sciences, Nanoscience and technology

## Abstract

The colloidal stability of air nanobubbles (NBs) was studied at different temperatures (0–30 °C) and in the presence of sulfates, typically found in mining effluents, in a wide range of Na_2_SO_4_ concentrations (0.001 to 1 M), along with the effect of surfactants (sodium dodecyl sulfate), chloride salts (NaCl), and acid/base reagents at a pH range from 4 to 9. Using a nanobubble generator based on hydrodynamic cavitation, 1.2 × 10^8^ bubbles/mL with a typical radius of 84.66 ± 7.88 nm were generated in deionized water. Multiple evidence is provided to prove their presence in suspension, including the Tyndall effect, dynamic light scattering, and nanoparticle size analysis. Zeta potential measurements revealed that NBs are negatively charged even after two months (from − 19.48 ± 1.89 to − 10.13 ± 1.71 mV), suggesting that their stability is due to the negative charge on their surface. NBs were found to be more stable in alkaline solutions compared to acidic ones. Further, low amounts of both chloride and sulfate dissolved salts led to a reduction of the size of NBs. However, when high amounts of dissolved salts are present, NBs are more likely to coalesce, and their size to be increased. Finally, the investigation of the stability of air NBs at low temperatures revealed a non-monotonic relationship between temperature and NBs upon considering water self-ionization and ion mobility. This research aims to open a new frontier towards the application of the highly innovative NBs technology on the treatment of mining, mineral, and metal processing effluents, which are challenging aqueous solutions containing chloride and sulfate species.

## Introduction

Nanobubbles (NBs), also known as ultra-fine bubbles, are defined as gaseous cavities with a diameter of less than 1 μm (typically less than 200 nm)^1–3^. They can be categorized as surface NBs, i.e., generated at solid–liquid interfaces, or bulk NBs, i.e., formed in bulk liquids^4,5^. Nanobubbles have many applications^6^ from wastewater treatment, ecosystem restoration, surface cleaning, oxygenation in agriculture and in aquaculture to disinfection, and medicine, including industrial applications^7^ that could be used in the current decarbonization efforts^8^ and the transition of our modern societies towards low-carbon electric economies. Nanobubbles are commonly generated by cavitation, which occurs when pressure falls below a particular threshold value, typically via acoustic, optical, particle, or hydrodynamic pressure reduction techniques^9–16^. Furthermore, sonochemistry using ultrasound^17^, electrolysis^18^, and applying nanopore membranes^19^ have been used to generate NBs. Fundamentally, the internal pressure of a bubble grows substantially as its diameter decreases, according to the Young–Laplace equation [Eq. (1)]^20,21^:

1 $${\mathrm{P}}_{\mathrm{i}}={\mathrm{P}}_{\mathrm{a}}+\frac{4\upsigma }{\mathrm{d}}$$

where P_i_, P_a_, σ, and d represent the internal pressure of the bubble, the atmospheric pressure, the surface tension of the liquid, and the bubble diameter, respectively. For instance, NBs with a radius of 100 nm have a high internal pressure of 1.56 MPa, according to the Eq. (1), while according to Oh et al^4^, the internal pressure of NBs (d = 88.50 nm and σ = 0.073 N/m) is approximately 3.4 MPa. As a result, the bubbles’ lifetime is thought to be extremely short (microseconds to milliseconds), as a high internal gas pressure should cause the gas inside the bubble to dissolve instantly into the bulk solution. Ljunggren et al.^22^ determined the lifetime (t_b_) of a bubble using the Eq. (2):

2 $${\mathrm{t}}_{\mathrm{b}}=\frac{{\mathrm{Kd}}_{0}^{2}}{12\mathrm{RTD}}$$

where d_0_, T, K, R, and D are the bubble diameter at time zero, temperature, Henry law constant, gas constant, and diffusion coefficient, respectively. For instance, NBs with size of 88.5 nm should exist for only 0.41 μs according to Eq. (2) ^4^. However, NBs exist and have been proven to be generally stable for prolonged periods of time in aqueous solutions^23^.

Various theories have been proposed to explain the experimentally observed stability of nanobubbles after their generation inside the solutions. For example, one theory has proposed that nanobubbles rely on Brownian motion to propel themselves instead of rising to the free surface as the result of a rather insignificant buoyant force^1,5^. Others have theorized that nanobubble stability is caused by their interfacial composition and structure^5^, which contains strong hydrogen bonds thus reducing the internal gas diffusivity^24^. Wang et al.^25^, based on spectroscopic force measurements, discovered that surface of NBs is kinetically stable even under high internal pressures, and that the gas–water interface has a high diffusive resistance. Also, experimental results have proven that NBs in water are negatively charged^26^, thus explaining their apparent stability. The latter can be supported by the electrically charged liquid–gas interface, which creates repulsive forces that prevent bubble coalescence^27^. In other words, the electric double layer prevents gas diffusion and bubble agglomeration^28–30^. Moreover, certain investigations have suggested that the internal gas pressure within nanobubbles might be lower than initially anticipated by Young–Laplace Eq. ^3,29^. For instance, electrostatic repulsion decreases the Young–Laplace pressure [Eq. (3)]; however, there is not enough pressure to balance out the Young–Laplace pressure^30^.

3 $$\Delta \mathrm{P}=\frac{4\upsigma }{\mathrm{d}}-\frac{4\upvarepsilon {\upzeta }^{2}}{{\mathrm{d}}^{2}}$$

where ε is the dielectric constant.

Another theory proposes that the surface adhesion of organic compounds or other amphiphilic particles and contaminants within a liquid, after NBs generation, has the potential to reduce surface tension and enhance the stability of NBs. Each nanobubble is coated with a trapped layer of insoluble contaminants. This leads to a decrease of the interfacial tension and the Young–Laplace pressure and creates a diffusion barrier^31^. Sugano et al.^32^ experimentally studied the effect of organic materials on NBs by transition electron microscopy (TEM) and dynamic light scattering (DLS) and found that organic materials adhered to the surface of NBs. Hence, the disappearance of NBs from the solution is prevented and NBs stability is improved. Shi et al.^29^ estimated the internal pressure of different gaseous NBs, including N_2_, O_2_, H_2_, and CO_2_, in water based on a model developed using a modified Young–Laplace equation that accounts for colloidal forces at the NBs water–gas interface. They reported NBs internal pressures in the range of 0.83–1.65 MPa, which were lower than the internal pressures estimated by the Young–Laplace equation. However, the internal pressure is still higher than atmospheric pressure, which should theoretically make the long-term existence and stability of NBs impossible. As a result, the colloidal lifetime and stability of NBs in liquid are still widely debated topics.

Although none of the proposed theories responds to all cases, NBs applications continue to attract significant attention from the scientific community due to their unique physicochemical properties, such as their stability and reactivity. To-date, NBs have found applications in several fields, including agriculture and animal husbandry^33^, fuels combustion^34^, wastewater and drinking water treatment^15,35,36^, water recycling^7,35^, surface cleaning^7^, sediment decontamination^37,38^, and mining and mineral processing^35,39^. The mining industry has a lot to gain from NBs technology, not only in mineral processing (i.e., froth flotation) and/or advanced oxidant delivery applications in hydrometallurgy, but also regarding its ability to efficiently recycle water in its operations in a clean sustainable fashion. However, to-date, the existence and stability of NBs has not been studied at low temperatures (from 0 to 10 °C), which are often found in cold regions where mining operations often take place, nor in the presence of sulfates, which is a common reagent category used in mineral and metal processing.

In this paper, the colloidal stability of air NBs at low temperatures and in the presence of sulfates was experimentally investigated. The results of this study expand on our current knowledge and understanding of the stability of NBs in aqueous solutions under well-defined conditions, which are essential to develop applications that deal with aqueous wastes from the mining, mineral, and metal processing industry. The long-term stability of bulk NBs suspensions was assessed throughout a period of 2 months. An additional innovation within this research is studying the behavior of NBs at a wide range of chloride and sulfate concentrations, providing insights into the mechanisms of interactions between salts and NBs. Also, the effect of pH and presence of surfactants on the physicochemical characteristics of NBs were studied. This study represents an effort in examining the stability of NBs under cold temperature conditions, specifically below 10 °C and proposed possible mechanisms that explain the effect of temperature on NBs stability. The effect of low temperatures and sulfates investigated in this work provides key insights that promise to unlock the potential of NBs technology in environmental applications for the development of next generation extractive processes, such as mining, mineral, and metal processing. The data reported in this work can be essential to develop novel NBs-driven applications to treat mining, mineral, and metal processing effluents and provide solutions that will help the industry attain zero liquid discharge processing.

## Methodology

### Materials

Sodium chloride (NaCl, 99.6%), sodium sulfate (Na_2_SO_4_, 99%), and sodium dodecyl sulfate (SDS, > 99%) were purchased from Fisher Scientific. Sodium hydroxide (NaOH, 1 M), hydrochloric acid (HCl, 37%), and sulfuric acid (H_2_SO_4_, 95–98%) were purchased from VWR Chemicals and were used (as stock solution at 0.5 M) to adjust the pH of the test solutions used in this study. All stock solutions and NBs samples were prepared using deionized water (DI). Air was used to generate the NBs.

### NBs generation

An MK1 Nanobubbler™ (Fine Bubble Technologies Ltd., Cape Town, South Africa), built with stainless-steel grade 316, was used for 60 min at an air flow rate of 170 L/min to generate air NBs in DI water. Air molecules are first sucked by the nanobubbler, passing through a venturi which generates microbubbles in the water. The microbubbles/water mixture is then fed into a cavitation cylinder which generates nano-size bubbles in the aqueous solution. After NBs generation, the solutions of NaCl, Na_2_SO_4_, and SDS were prepared by dissolving appropriate amounts of NaCl, Na_2_SO_4_, and SDS in water containing NBs using a magnetic stirrer at 20 °C until complete dissolution was achieved. All the samples were stored in sterilized plastic tubes at ambient conditions.

### Experimental procedure to assess air NBs stability

Upon the generation of the NBs suspensions, samples were collected and stored in 40 mL tubes for the analysis of bubble size distribution, concentration, zeta potential, and long-term stability. All NBs samples were stored and analyzed at room temperature with the exception of a limited number of samples, which were used to study the effect of temperature on NBs. For the latter, solution samples containing NBs were generated at room temperature then placed in a fridge at 0, 5 and 10 °C, and on a hot plate at 30 °C for 1 day in order to be stabilized and immediately analyzed. To ensure that the samples were prepared at a temperature close to 0 °C and to prevent the water from freezing, the temperature was carefully controlled and maintained near the freezing point. The conditions and compositions of the samples tested, including the concentration of the NaCl, Na_2_SO_4_, and SDS, are shown in Table 1.

**Table 1:** Conditions and compositions of the air NBs-containing aqueous solutions used in this work.

Set of conditions	Time	pH	T (°C)	C_NaCl_ (M)	$\text{C}_{\text{Na}_2\text{SO}_4}$	C_SDS_/CMC*
1	0 to 8 weeks	6.7	20	–	–	–
2	1 day	4–9	20	–	–	–
3	1 day	6.7	0, 5, 10, 20 & 30	–	–	–
4	1 day	6.7	20	0.001, 0.01, 0.1, 0.5 & 1	–	–
5	1 day	6.7	20	–	0.001, 0.01, 0.1, 0.5 & 1	–
6	1 day	6.7	20	–	–	0.5, 1, 2, 3, 4, 5, 6 & 8

*Critical micelle concentration (CMC) = 8.2 × 10^–3^ M.

### Particle size distribution

#### Dynamic light scattering (DLS)

A DLS technique (NanoBrook Omni, Brookhaven Instruments Corporation, USA) was employed for the quantification of the bubble size distribution of the NBs samples collected in this work. DLS is an advanced non-invasive method for determining particle size distributions, which is typically used to estimate particles, emulsions, or molecules dispersed in a liquid. Laser light is scattered at different intensities due to the Brownian motion of particles or molecules in suspension^5^. Therefore, these intensity fluctuations can be converted to Brownian motion velocity and hence particle size using the Einstein–Stokes equation [Eq. (4)]^5,40^.

4 $$\frac{\overline{{(\mathrm{x},\mathrm{ y})}^{2}}}{4\mathrm{t}}={\mathrm{D}}_{\mathrm{t}}=\frac{{\mathrm{k}}_{\mathrm{B}}\mathrm{T}}{3\mathrm{\pi \mu d}}$$

where $\overline{{(\mathrm{x},\mathrm{ y})}^{2}}$ represents the displacement of a nanoparticle in two dimensions measured over time t, and D_t_, k_B_, T, $\upmu$, and d correspond to the diffusion coefficient, Boltzmann constant, temperature, viscosity, and particle diameter, respectively. DLS can analyze particles with sizes ranging from 0.3 nm to 10 $\upmu$m^41^. However, DLS does not quantify bubble concentration. In this work, a polystyrene latex standard with a size of 92 ± 3 nm was used to calibrate the instrument. The equipment was configured at a 90-degree angle. Following a stabilization period of 10 min, a total of ten measurements, each lasting 120 s, were conducted for every sample. The outcomes were denoted as the effective radius. The analysis employed Eppendorf disposable plastic cuvettes (#952010051), obtained from Mississauga, ON, Canada. To handle the size distribution, the CONTIN algorithm was employed.

#### Nanoparticle tracking analysis (NTA)

Nanoparticle tracking analysis (NTA) (ZetaView® BASIC NTA, Particle Metrix, DE) was used to measure bubble size distribution and concentration. NTA is a non-invasive technique that utilizes both the properties of light scattering and Brownian motion of dispersed particles to obtain particle size distributions of bubbles in solution. This is accomplished via a laser beam passing through a chamber containing the sample. This beam scatters light upon incident bubbles, which can be detected through the use of a long working distance microscope objective installed on an otherwise conventional optical microscope^5^. A video is captured using high-sensitivity cameras attached to the microscope to visualize bubbles moving under Brownian motion. Consequently, NBs are indirectly tracked, and their Brownian motion is analyzed in real time to determine their mean diameter and density. NBs are tracked in 2D to determine the diffusion coefficient of Brownian motion using Eq. (4). NTA is used primarily to analyze systems with particle sizes ranging from 10 to 2000 nm and concentration of 10^6^ to 10^9^ particles/mL^5^ and therefore, is directly applicable to the samples collected in this work. The instrument was cleaned according to manufacturer's instructions and calibrated using a 250,000-fold dilution of Nanosphere Standards (Thermo Scientific, #3100A) in ultrapure water (18.2 MΩ cm). Measurements for the 250,000-fold dilution of Nanosphere Standards were conducted with the following parameters: Sensitivity = 70, Shutter = 50, and Frame rate = 30 frames per second. Following data acquisition, the standard was analyzed using the following settings: Maximum Area = 10,000, Minimum Area = 1, and Frame Minimum Brightness = 20.

### Zeta potential and pH

Zeta potential measurements were carried out to determine the stability of NBs in solution, using BI-SCP disposable plastic cuvettes and a BI-ZEL electrode assembly from Brookhaven Instruments Corporation. All measurements were conducted using a wavelength of 640 nm. Zeta potential was measured based on one of the electrokinetic effects namely electrophoresis. In the electrophoresis technique, the zeta potential is determined by exposing fine particles to an electric field and measuring their mobility, indicated as υ_E_, through a suitable microscopic method^42^. The relationship between the mobility and the zeta potential at the interface is established through the utilization of the Smoluchowski equation [Eq. (5)]^42^:

5 $${\upsilon }_{E}=4\pi {\varepsilon }_{0}{\varepsilon }_{r}\frac{\zeta }{6\pi \mu }(1+\kappa r)$$

where, ζ, ε_0_, and ε_r_ represent the zeta potential, the relative dielectric constant, and the electrical permittivity of vacuum, respectively; μ stands for the viscosity of the solution; r represents the radius of the particle; κ is defined as (2n_0_z^2^e^2^/ε_r_ε_0_k_B_T)^1/2^ and it is known as the Debye–Hückel parameter. In this context, n_0_ signifies the bulk ionic concentration, z corresponds to the ion's valence, e is the charge of an electron, k_B_ represents the Boltzmann constant, and T indicates the absolute temperature. The pH of the samples was measured using a pH meter (Thermo Scientific), which was calibrated by standard buffer solutions at 20 °C.

## Results and discussion

### Generation and stability of NBs

**Figure 1:**
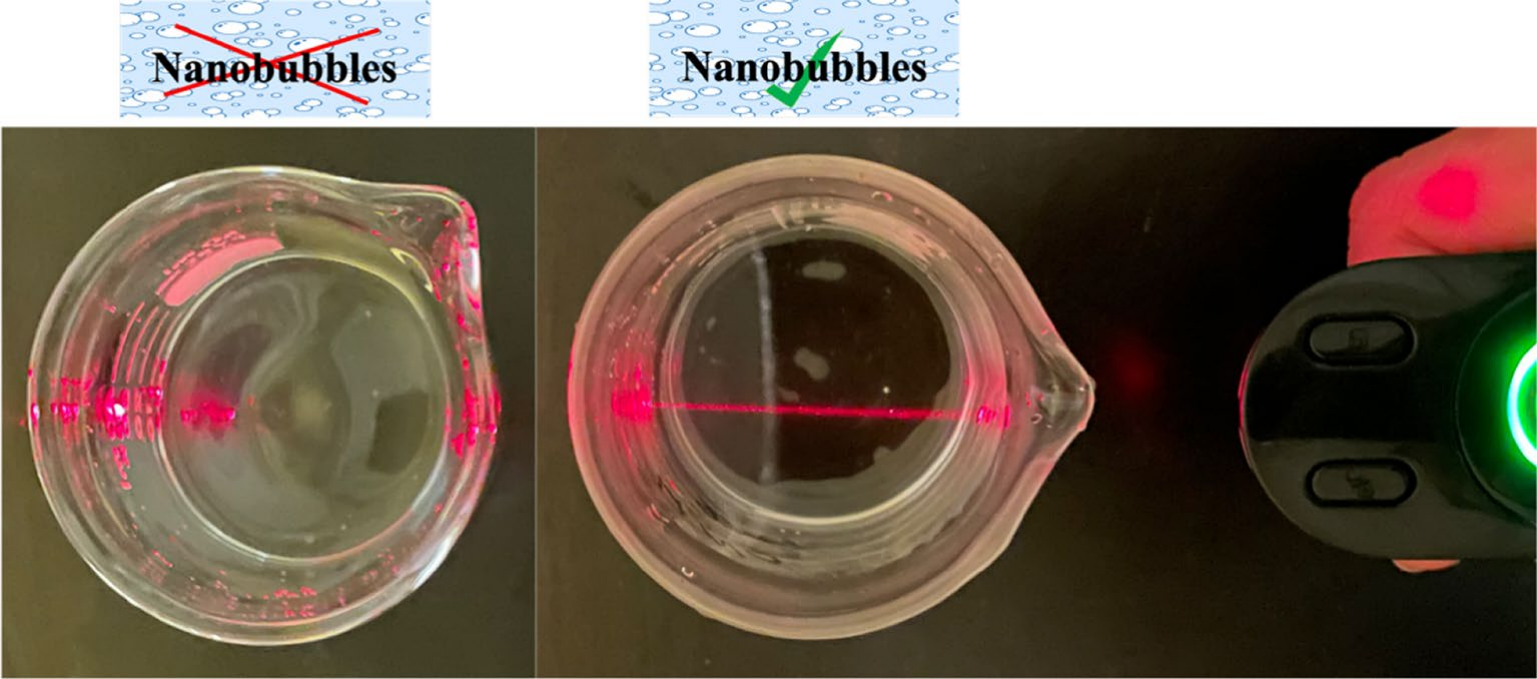
The Tyndall effect proves the presence of NBs: a laser beam is scattered in a solution containing NBs allowing the laser pathway to be detected.

NBs were fabricated using an MK1 Nanobubbler™ with air and DI water. The presence of NBs in water was first proven using a visual verification method, namely the Tyndall effect (Fig. 1). This is a well-established phenomenon in colloidal solutions that can be used to test whether nanobubbles exist in aqueous solutions: a laser beam is scattered in a solution containing NBs, while the light path from the same laser beam cannot be detected in a water sample without the presence of NBs^43^. During our experiments, no contaminants were introduced, suggesting that the colloid particles were indeed air nanobubbles, which is consistent with observations that have been reported in the literature^43–46^.

**Figure 2:**
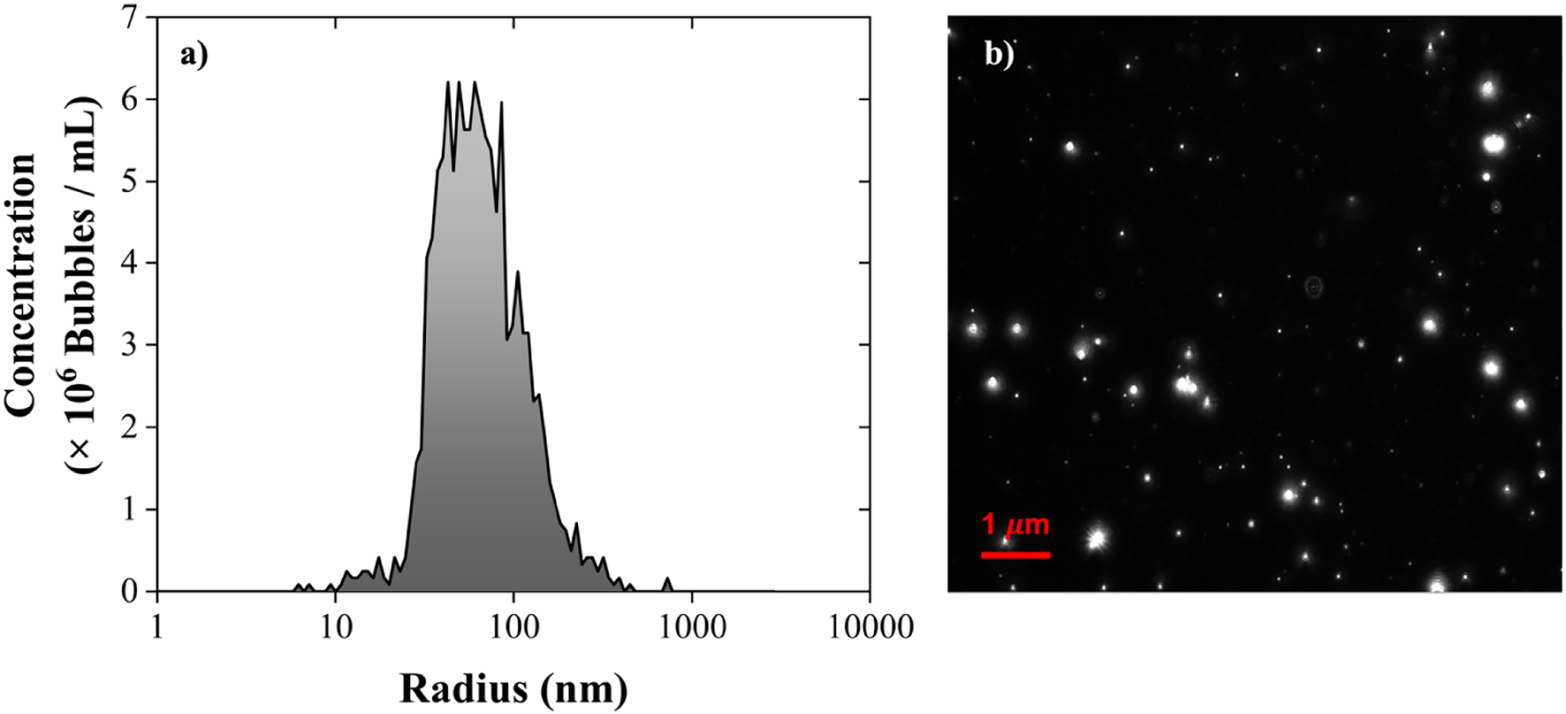
NBs analyzed on the same day of their generation (**a**) NTA size distribution and (**b**) micrograph of the NBs suspension in solution.

Figure 2 shows the NBs size distribution as well as a micrograph of the NBs obtained in the same day of the NBs generation. The white dots in the black background represent the NBs; their concentration was measured at 1.2 × 10^8^ bubbles /mL and their mean radius at 84.66 ± 7.88 nm. No nano-size impurities were found in the blank sample containing DI water prior to the NBs generation. Numerous studies on NBs generation suggest a consistent concentration of approximately 10^8^ bubbles/mL at room temperature, regardless of operational variables or conditions^4,47^. There are some plausible explanations that could contribute to this point. For example, the generation and persistence of NBs could reach a thermodynamic equilibrium point under common ambient conditions, leading to a relatively consistent concentration across different setups. Furthermore, the solubility of gases in water at typical temperatures might set a limit on the concentration of NBs that can form before reaching saturation. Also, the balance between surface tension and gas pressure within the NBs could reach a point where further increase in concentration is limited to maintain bubble stability. Finally, the kinetics of bubble nucleation and growth might be a dominant factor, resulting in a self-regulated concentration regardless of external conditions.

**Figure 3:**
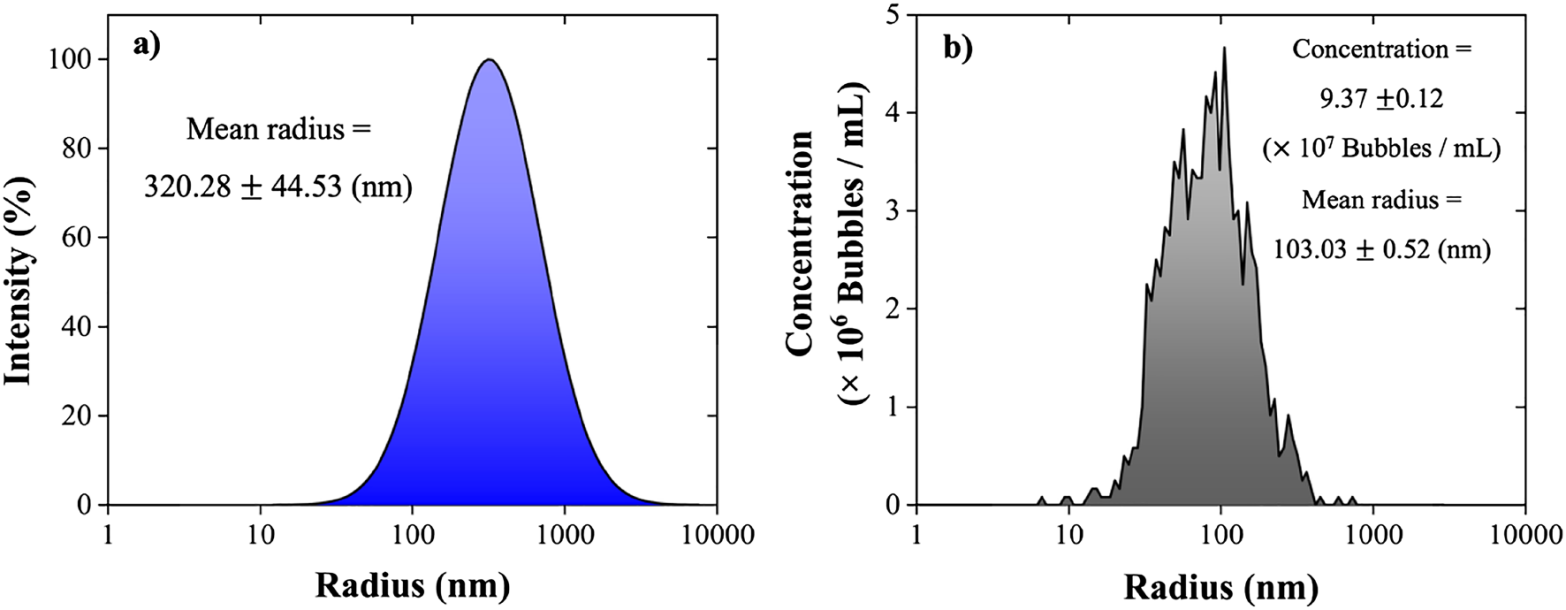
Air NBs size distribution 1 day after their generation obtained by (**a**) DLS and (**b**) NTA.

After 1 day from the NBs generation, a single narrow peak value detected by DLS, which corresponds to a mean NBs radius of 320.28 ± 44.53 nm (Fig. 3a). The NTA results for the same sample translate to a NBs concentration of 9.37 ± 0.12 (× 10^7^ bubbles/mL) and a mean radius of 103.03 ± 0.52 nm (Fig. 3b). An obvious shift in the bubble size distribution can be detected by comparing the DLS and NTA results: the mean radius of the NBs measured by NTA is consistently lower than the one obtained by DLS. The reason is that DLS works based on the intensity of scattered light, which generates results that are biased by larger bubbles^5,48,49^. This shift in the NBs size measurements between DLS and NTA has also been reported in literature^5^.

**Figure 4:**
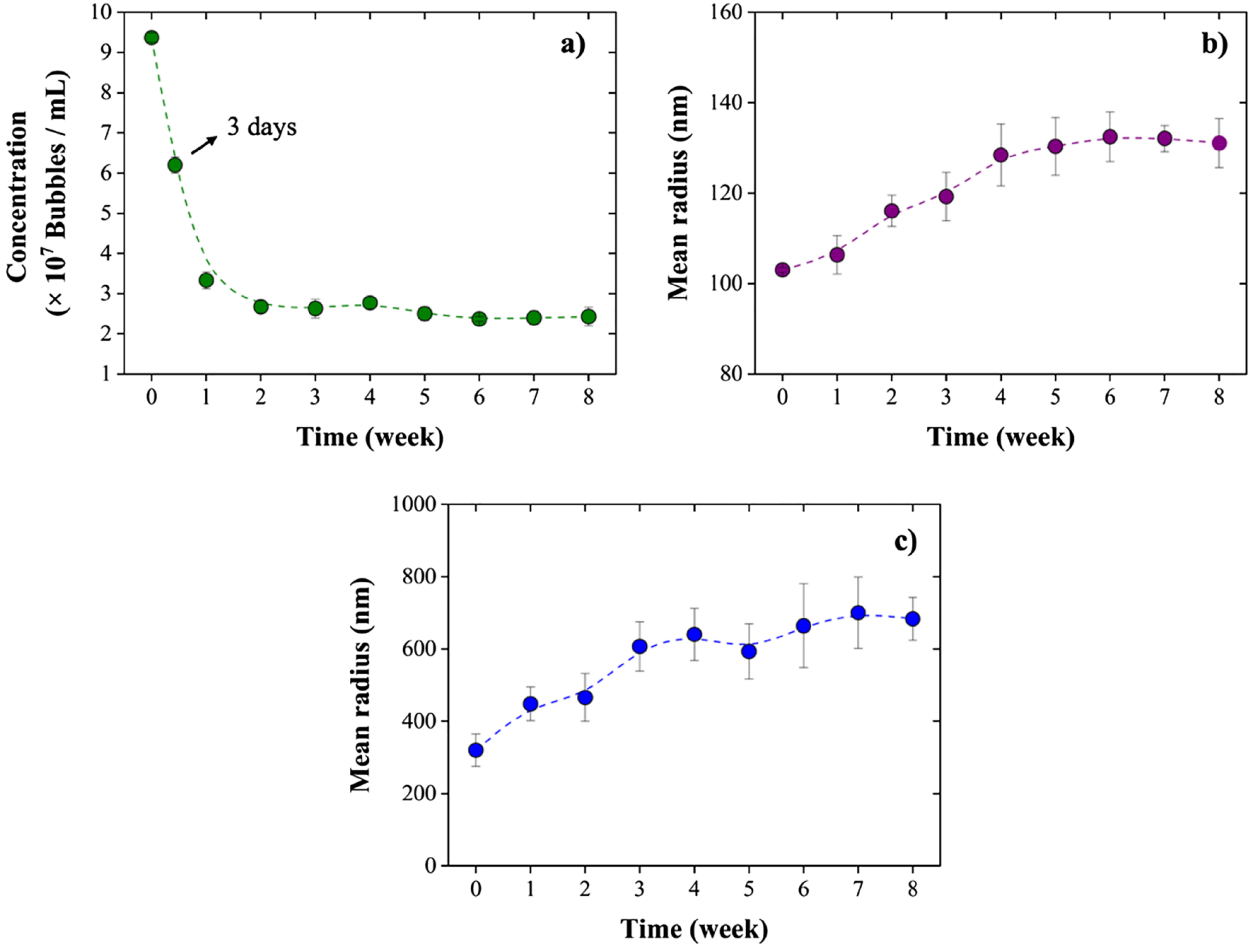
Stability of NBs from 1 day until up to 8 weeks after their generation: (**a**) size of NBs measured by NTA and (**b**) concentration of NBs measured by NTA, and (**c**) NBs size measured by DLS.

**Figure 5:**
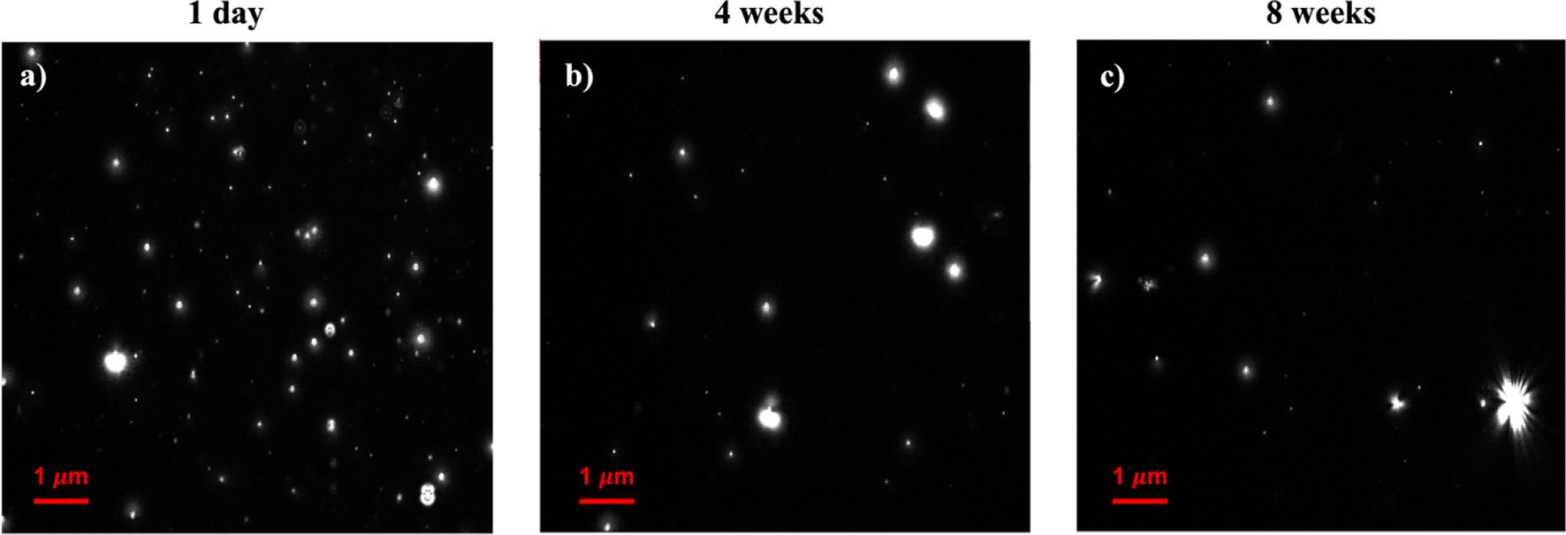
NTA micrographs of NBs after (**a**) 1 day, (**b**) 4 weeks, and (**c**) 8 weeks from the time of their generation.

Among the most extraordinary characteristics of NBs is their unusually long lifespan, with NBs being stable in solution for several days^33,50^. Here, we monitored the stability of air NBs in DI water. Figure 4 presents the NBs stability results for up to 8 weeks after their generation, using both NTA and DLS. Accordingly, NBs were found to be stable even after 8 weeks; however, their concentration decreased by approximately 74% over this 8-week period: from 9.37 × 10^7^ bubbles/mL (± 1.28%) 1 day after their generation to 2.43 × 10^7^ bubbles/mL (± 9.46%) after 8 weeks (Fig. 4a). The mean radius of NBs increased by 27.22% after 8 weeks, reaching 131.08 ± 5.42 nm (compared to their initial 103.03 ± 0.52 nm radius), according to the NTA results presented in Fig. 4b. The results obtained from the DLS analyses also confirm the increase in the size of NBs (around 2.5 times) as indicated in Fig. 4c. Bubbles that continue to exist in solution are more prone to experiencing coalescence over time. As a result, the bubbles can agglomerate leading to an increase in their size. Figure 5 shows the NBs micrographs on the first day from the NBs generation, after 4 weeks, and after 8 weeks, which provides a visual representation of the disappearance of the bubbles. It is obvious that the number of NBs decreases over time; however, NBs were still present in the samples in significant amounts even after 8 weeks.

Similarly, other researchers have shown that NBs remain in water for prolonged periods of time. Nirmalkar et al.^5^ generated NBs in DI water by acoustic cavitation and analyzed their samples using NTA. Their reported NBs mean diameter and concentration were 100–120 nm and 100 × 10^7^ bubbles/mL, respectively. Further, they found that 30–50% of the NBs remained in their samples after 50 days. Moreover, Phan et al.^51^ detected CO_2_ NBs in DI water even 7 days after their generation using DLS. They reported that the size of CO_2_ NBs right after generation and 7 days later were 591 ± 94 and 429 ± 122 nm, respectively. Accordingly, the size did not change significantly, thus indicating the high stability of the NBs generated. However, NBs being stable in water for a long time contradicts the theoretical knowledge. As such, alternative ideas for the existence and stability of NBs in solution have been suggested. For instance, no net diffusion of gas can occur at the gas–water interface due to the formation of an electric double layer around the NBs^15,50^. This electrical charge prevents bubble coalescence by repelling them from one another^44,52^. Moreover, the presence of hard hydrogen bonds on NBs interfaces, which helps stabilize the NBs, has been revealed by infrared spectroscopy measurements^24^. Therefore, NBs stabilize themselves in solution via ionic and diffusive shielding^4,52^.

**Figure 6:**
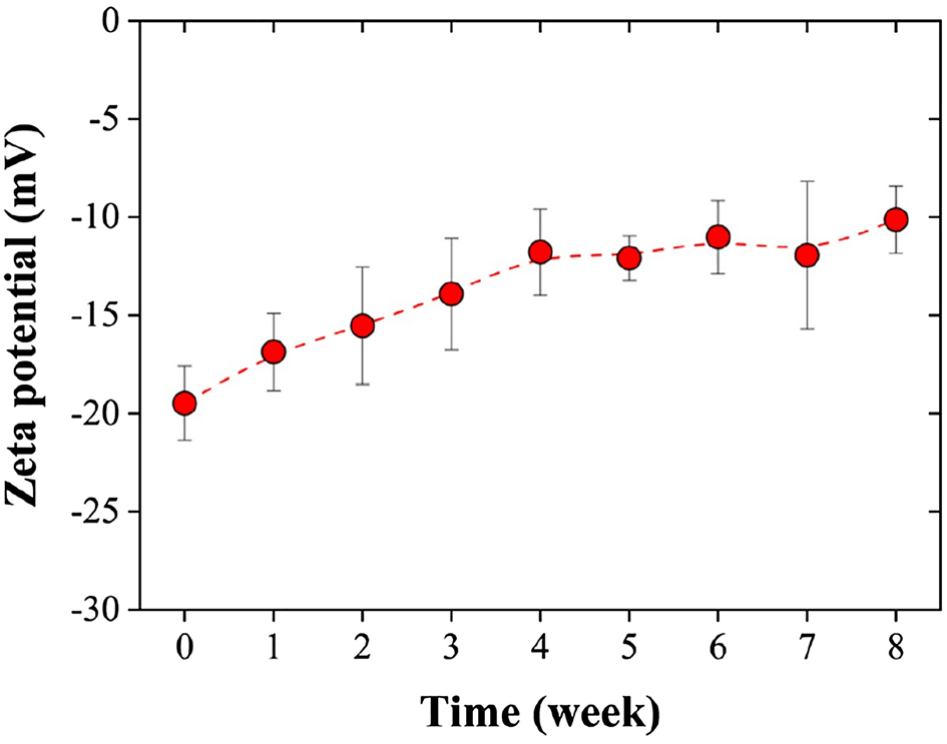
The zeta potential of air NBs over 8 weeks: its absolute value decreases but remains strongly negative. The negativity of the zeta potential indicates the high stability of the generated NBs.

In NBs, the negative electrical charge may be due to hydroxyl ions being attracted to the gas–water interface. It has been suggested that the orientation of water dipoles at the interface leads to possessing the dangling O–H group at a certain angle to the interface^5,44^. The zeta potential of the air NBs in this work was − 19.48 ± 1.89 mV at the first day of their generation and − 10.13 ± 1.71 mV after 8 weeks (Fig. 6), proving the stability of NBs. These results are consistent with literature: the zeta potential of air NBs in a pH range between 5.7 and 7 has been reported to range from approximately − 28 to – 20 mV^5,53^. According to Fig. 6, the absolute value of zeta potential for the NBs that remained in solution decreased over time, meaning that the repulsive forces between NBs were weakened, which enhances the bubble coalescence and their size.

### Effect of pH on NBs

**Figure 7:**
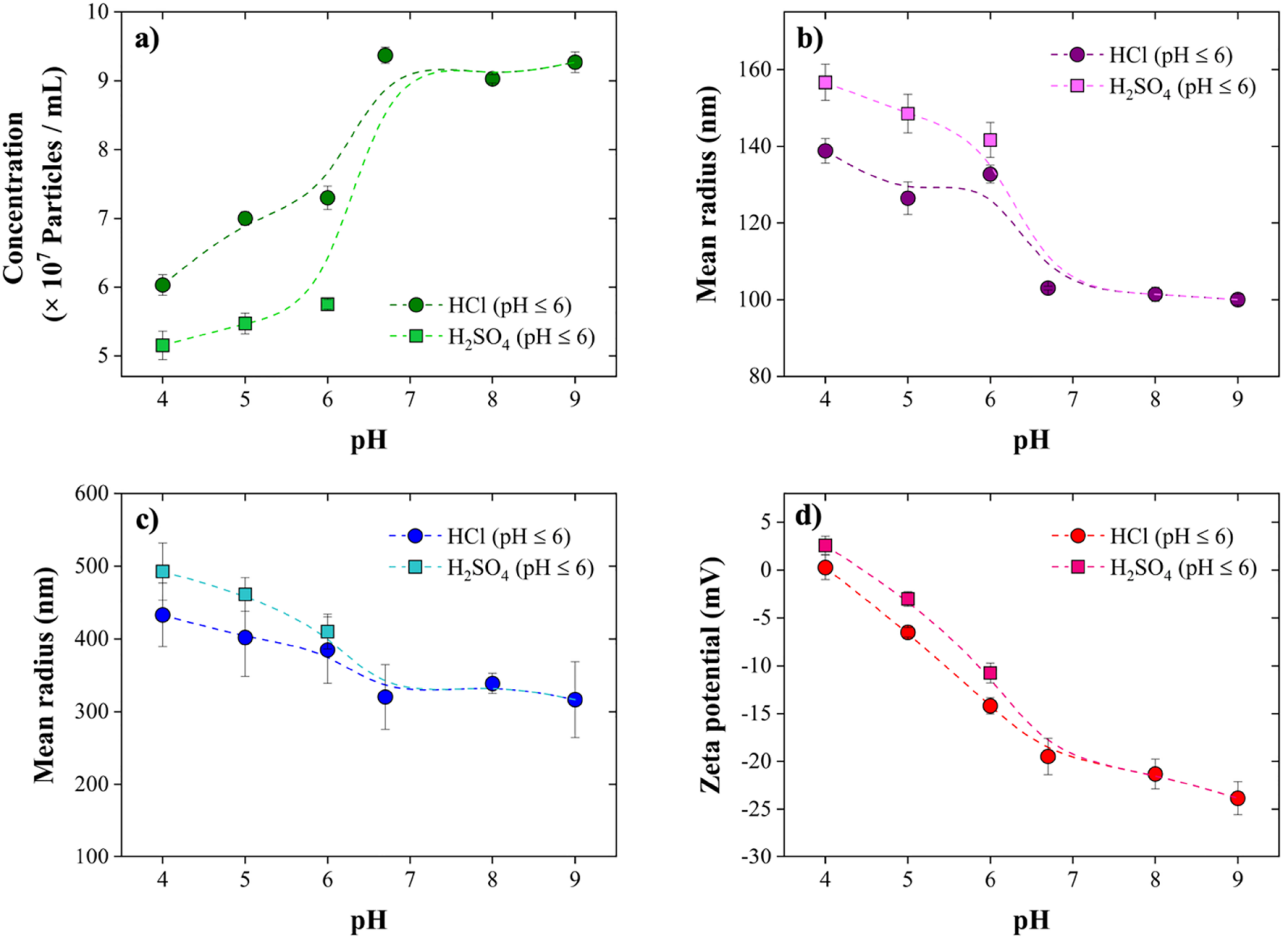
Effect of pH on the stability of NBs: (**a**) concentration and (**b**) mean radius of NBs obtained by NTA, (**c**) NBs mean radius obtained by DLS, and (**d**) zeta potential of NBs. Both the concentration and the absolute value of the zeta potential of NBs increase with increasing pH, while their size decreases, indicating that alkaline solutions provide more favorable conditions for the stability of NBs.

The effect of solution pH (HCl and H_2_SO_4_ for acidic range) on the NBs’ size (i.e., mean radius), concentration, and zeta potential is presented in Fig. 7. Specifically, Fig. 7a shows that the concentration of NBs was not significantly affected when increasing the pH from 6.7 to 9 with the reported NBs concentration and radius being relatively stable at 9.27 ± 0.15 (× 10^7^ bubbles/mL) and 100.03 ± 1.39 nm, respectively. However, the NBs concentration was decreased approximately 35.64% and 45.04% once pH was decreased from 6.7 to 4 by HCl and H_2_SO_4_ (Fig. 7a). Also, according to NTA analyses, the mean radius of NBs increased from 103.03 ± 0.52 nm to 138.85 ± 3.19 nm and 156.68 ± 4.68 nm by decreasing pH from 6.7 to 4 by HCl and H_2_SO_4_, respectively (Fig. 7b). Moreover, the increase in the mean radius of NBs was confirmed by DLS results (Fig. 7c): the mean radius was increased from 320.28 ± 44.53 to 433.09 ± 43.72 nm (upon adjusting the pH with HCl) and 492.76 ± 39.27 nm (upon adjusting the pH with H_2_SO_4_). Overall, NBs size tends to decrease and their concentration to increase with increasing pH due to the availability of OH^−^ ions, which are required to stabilize the electric double layer around the bubble interfaces^5,9^. This theory was confirmed by the zeta potential measurements shown in Fig. 7d. Accordingly, the zeta potential value of the NBs was negative (− 19.48 ± 1.89 mV) at pH 6.7, and shifted to more negative values, namely − 23.84 ± 1.74 mV at pH 9, while at pH 4 the zeta potential was almost 0 in both HCl and H_2_SO_4_ solutions. The latter shows a decreased stability of NBs in acidic solutions mainly because of the lower concentration of OH^−^ ions at lower pH, causing the repulsive forces between the NBs to decrease and the bubbles to coalesce, agglomerate, and break in the solution or escape from it. Hence, alkaline solutions provide more favorable conditions for stable NBs due to the stronger electrostatic interactions in solution. Furthermore, the comparison between the concentration, size, and zeta potential results obtained from HCl and H_2_SO_4_ solutions (Fig. 7a–d) indicates that H_2_SO_4_ provides harsher conditions for stable NBs. Lower concentration of NBs with larger size existed in the H_2_SO_4_ solutions compared to HCl ones. Also, the low absolute values of zeta potential confirm the relative instability of NBs in the presence of H_2_SO_4_. In fact, at the same pH value, less negative ions are accessible in H_2_SO_4_ solutions compared to HCl solutions, according to stoichiometry (0.5 SO_4_^2−^ vs. 1 Cl^−^). Additionally, the higher mobility of Cl^−^ than SO_4_^2−^ may contribute to a higher amount of Cl^−^ adsorption on NBs in HCl solutions than SO_4_^2−^ in H_2_SO_4_ solutions^54^.

### Effect of electrolyte concentration on NBs

**Figure 8:**
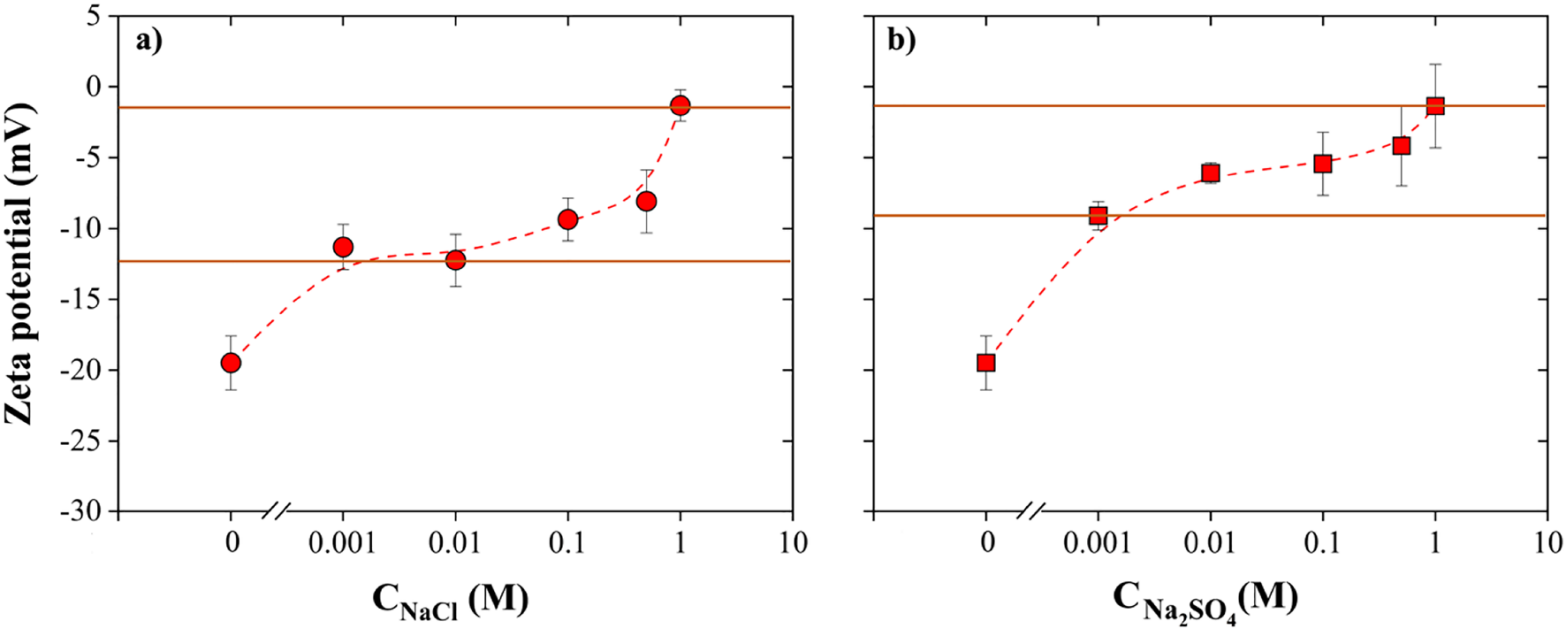
Effect of (**a**) NaCl and (**b**) Na_2_SO_4_ concentration on the zeta potential of air NBs (T = 20 °C, pH 6.7): increasing the dissolved salt concentration decreases the absolute value of NBs’ zeta potential, thus leading to decreased NBs stability.

Increasing salt concentration drives the zeta potential of NBs towards zero (Fig. 8). The effect of dissolved salts on the zeta potential of NBs can be explained by the Debye length (${\uplambda }_{\mathrm{D}}$), which represents how far the electrostatic effects of ions in electrolytes persist and can be calculated by Eq. (6) ^5^:

6 $${\uplambda }_{\mathrm{D}}=\sqrt{\frac{\upvarepsilon {\mathrm{k}}_{\mathrm{B}}\mathrm{T}}{{\mathrm{e}}^{2}\mathrm{I}}}$$

where $\varepsilon$, k_B_, T, and e correspond to the medium permittivity, Boltzmann constant, temperature, and elementary charge, respectively. The ionic strength (I) of the salt, which can be measured by Eq. (7) ^55^, increases when the salt concentration increases, leading to a decrease of the Debye length [Eq. (6)].

7 $$\mathrm{I}=0.5\sum {\mathrm{C}}_{\mathrm{i}}{\mathrm{z}}_{\mathrm{i}}^{2}$$

where z_i_ is the valance of ion i and C_i_ is the molar concentration of salt. However, the same concentration of Na_2_SO_4_ was found to decrease the absolute value of NBs’ zeta potential more than NaCl as indicated in Fig. 8a and b (p-value is 0.02% << 5%). This can be interpreted by the higher ionic strength of Na_2_SO_4_ compared to NaCl, which, according to Eqs. (6) and (7), leads to a reduction of the repulsive forces between NBs. Further, the fact that Cl^−^ has a higher mobility than SO_4_^2−^ may lead to a higher degree of adsorption of Cl^−^ at the NBs interface in NaCl solutions compared to that of SO_4_^2−^ in Na_2_SO_4_ solutions^54^. Moreover, more positive ions (Na^+^), two times more based on stoichiometry, are available in Na_2_SO_4_ solutions compared to NaCl solutions of the same concentration. As a result, the negativity of the NBs’ zeta potential is expected to be higher in NaCl solutions than Na_2_SO_4_ solutions.

**Figure 9:**
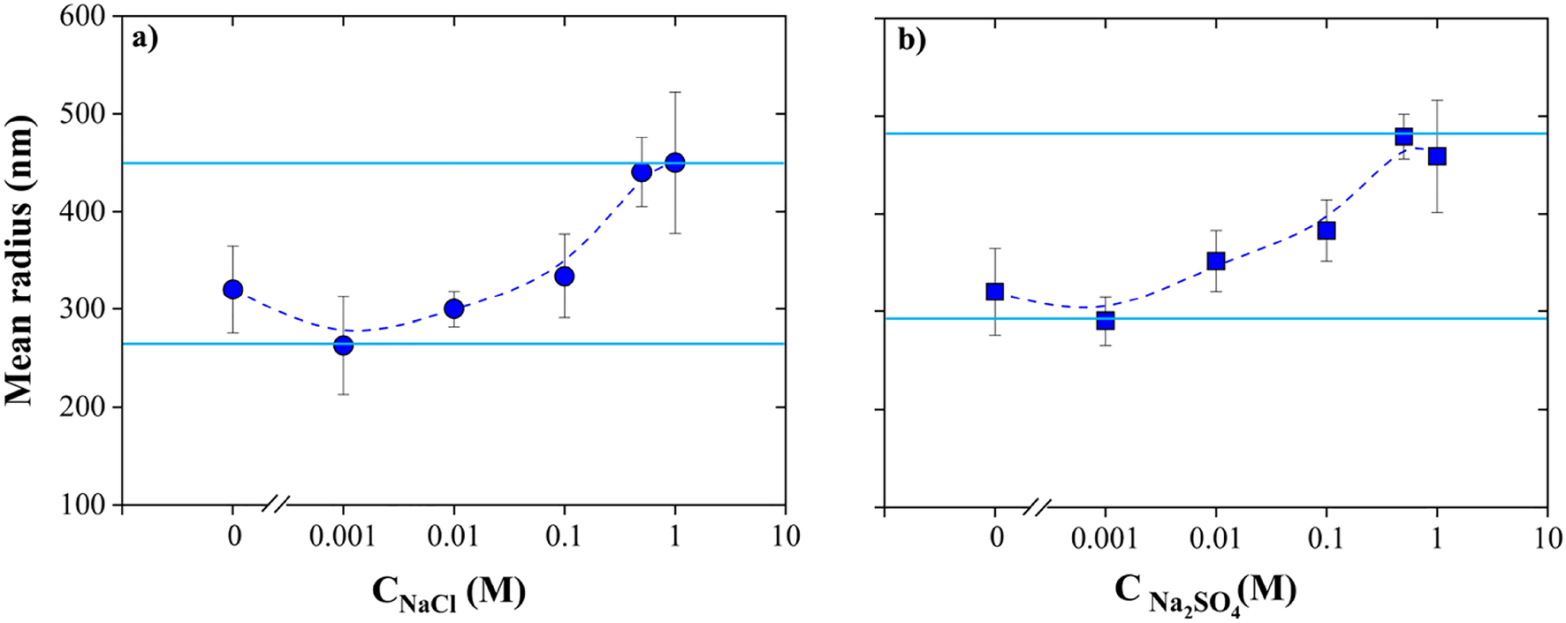
Effect of (**a**) NaCl and (**b**) Na_2_SO_4_ concentration on the mean radius of NBs (T = 20 °C, pH 6.7): the presence of low amount of dissolved salts leads to a decrease in the size of NBs, while a further increase in the dissolved salt concentration leads to an increase of the NBs.

Figure 9 indicates the impact of NaCl and Na_2_SO_4_ concentration on the mean radius of NBs. The effect of salt on the mean radius of NBs strongly depends on the concentration of dissolved salts in solution. As depicted in Fig. 9a and b, the size of NBs decreases upon the addition of low amounts of NaCl (0.001 M) and Na_2_SO_4_ (0.001 M) in water, followed by an increase of the air NBs size at higher NaCl and Na_2_SO_4_ concentrations (1 M). With a similar concentration of salt, the variation of NBs size for NaCl and Na_2_SO_4_ is influenced by the anion type, namely, Cl^−^ and SO_4_^2−^. The NBs in the Na_2_SO_4_ solutions tend to be larger compared to the NaCl solutions as depicted in Fig. 9b (p-value is 4.51% < 5%). As discussed earlier, the absolute value of zeta potential of NBs in sulfate solutions is lower than that in chloride solutions. Accordingly, weaker repulsive forces between NBs in sulfate solutions increase the probability of bubbles coalescence. Hence, larger bubbles exist in sulfate solutions compared to chloride ones. Agarwal et al.^56^ observed that the mean diameter of nanobubbles in a 0.001 M Na_2_SO_4_ solution is approximately 90 nm bigger than that of NBs in a NaCl solution of the same concentration. Further, Hewage et al.^54^ reported that the energy barrier to prevent bubble coalescence in a NaCl solution is 1.87 × 10^–20^ J, which is considerably higher than that in a Na_2_SO_4_ solution (near zero) based on the Derjaguin–Landau–Verwey–Overbeek (DLVO) calculations, which further indicates a higher degree of NBs instability in sulfate solutions. Also, in context of particle coagulation, solid particles generally aggregate more easily in Na_2_SO_4_ solutions compared to NaCl solutions. The valence of the ions in Na_2_SO_4_ is higher, which might lead to stronger ionic interactions compared to NaCl. Moreover, higher ionic strength can lead to stronger screening of electrostatic repulsions, potentially promoting particle aggregation^57^ and Na_2_SO_4_ has higher ionic strength compared to NaCl. As a result, solid particles aggregate easier in Na_2_SO_4_ solutions compared to NaCl solutions.

**Figure 10:**
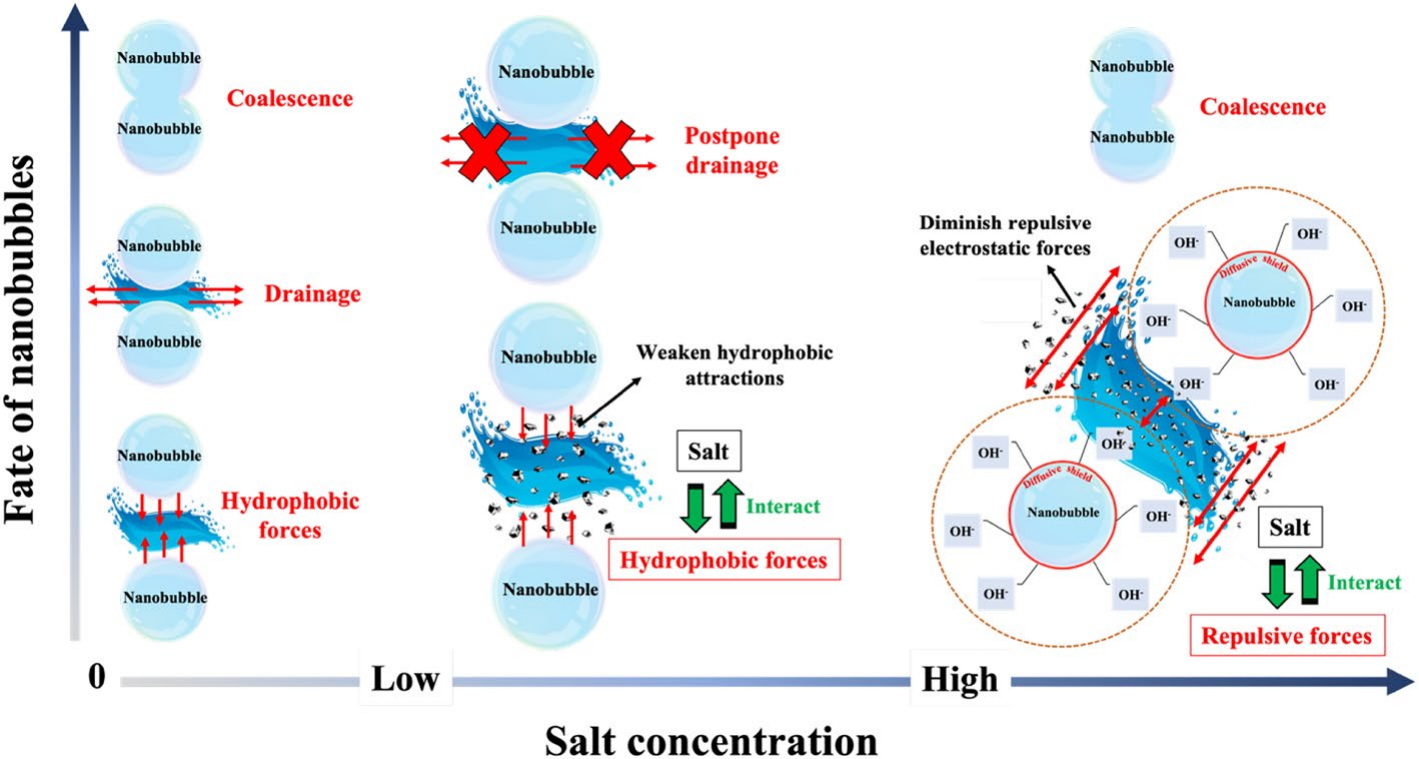
Dissolved salt concentration affect the size and stability of NBs according to two competing mechanisms: at low salt concentration, salts tend to inhibit bubble coalescence by weakening the hydrophobic attractions between NBs, while high salt concentrations tend to decrease the repulsive electrostatic forces between NBs thus leading to their coalescence.

Previous works primarily concentrated on investigating electrolytes that exhibit the capacity of bubble coalescence inhibition^58^. Firouzi et al.^59^ reviewed the influence of salt concentration on bubble coalescence and claimed that adding salts to water reduces bubble coalescence. According to the experimental results obtained by Craig et al.^58^, some salts have no effect on bubbles coalescence until reach to the transition concentration, which adding more salts causes inhibition effect. During the collision process between bubbles, it is evident that coalescence leads to a reduction in the total free energy by decreasing the area of the air–water interface^58^. The coalescence of bubbles in water necessitates the presence of a sufficiently robust attractive force between these bubbles. The main attractive force that operates between the surfaces of the bubbles is the long-range hydrophobic interaction^58^. In the case of bubbles, the inclusion of salts leads to a decrease in the range of the attractive force. It appears that these salts function to hinder the coalescence of bubbles by reducing the extent of attraction between them^58^. Also, the prevention of bubble coalescence could potentially be associated with the gas solubility within the electrolyte solution^60^. When a gas dissolves in a liquid, it can create internal pressure within the bubble due to the gas molecules accumulating at the liquid–gas interface surrounding the bubble. Salts can have an impact on this process and alter the gas solubility in the liquid. This is because salts can affect the properties of the liquid, such as its surface tension and molecular interactions, which in turn influence the solubility of gases. As a result, the presence of salts can lead to changes in the amount of gas that can dissolve in the liquid, affecting the internal pressure of the bubble. Weissenborn et al.^60^ experimentally showed that vapor cavities form upon contact between hydrophobic surfaces. There are indications that bridging cavities could potentially contribute to the observed long-range attraction. In this context, the addition of more salts (5 M NaCl) seems to do not lead to a decrease in the attraction^60^. Moreover, the presence of salts in solutions containing NBs also diminishes the repulsive electrostatic forces between them. This phenomenon tends to increase NBs coalescence. Hence, the effect of salt concentration on NBs ought to be interpreted upon considering both these effects, as indicated in Fig. 10. When the concentration of dissolved salts is low, the coalescence inhibition effect of salt overcomes the negative impact of salts on the repulsive forces. Consequently, low amounts of salts cause a decrease in the size of NBs. However, the probability of NBs coalescence at high amount of salt increases leading to NBs agglomeration, whereby their size increases. Similar trends have been observed in literature^5,9,61^. For instance, Xu et al.^62^ observed a reduction in bubble size with the addition of 0.25 wt% NaCl, while Meegoda et al.^9^ observed a NBs size increase and a reduction in their absolute zeta potential value in the presence of 1 M of NaCl. Also, according to Phan et al.^51^, the size of CO_2_ NBs increased from around 600 nm to around 900 nm upon the addition of 0.5 wt% NaCl; however, adding 0.1 wt% of NaCl did not change their size significantly.

### Effect of surfactant on NBs

**Figure 11:**
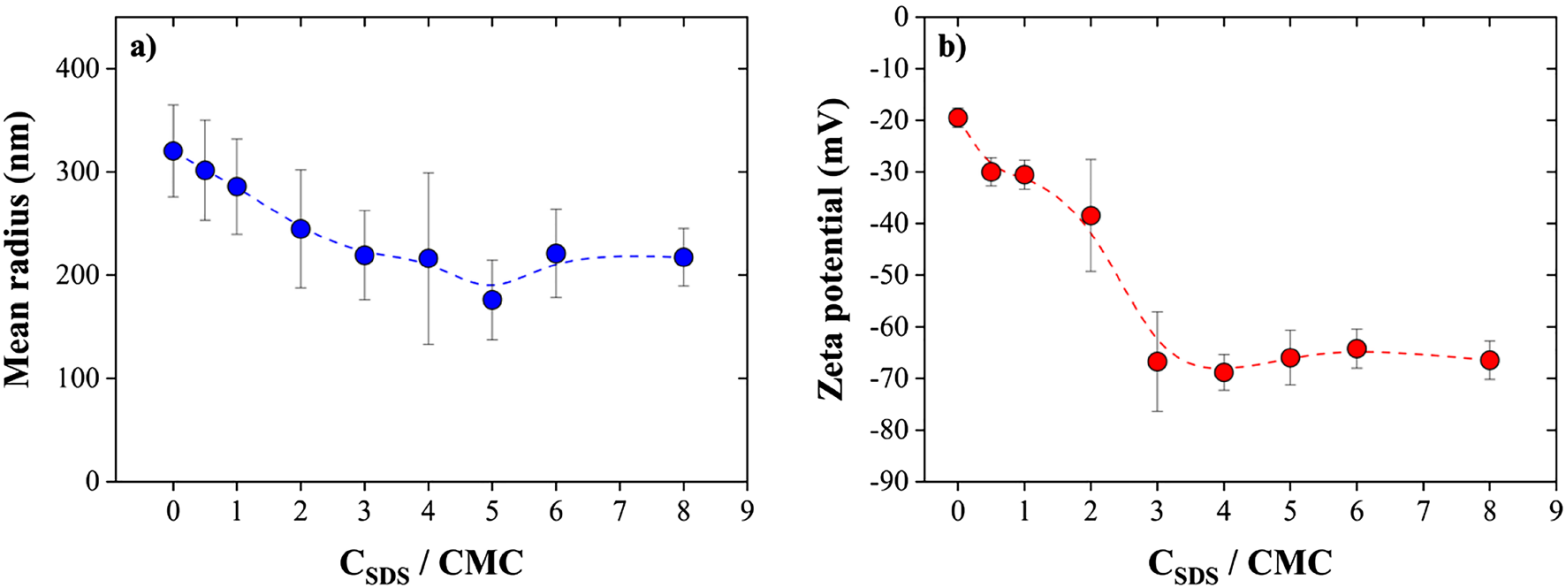
Impact of SDS concentration on the (**a**) mean radius and (**b**) the zeta potential of NBs (T = 20 °C, pH 6.7): the size of NBs decreases in the presence of SDS; however, the absolute value of zeta potential of NBs increases, which shows an improved stability of NBs in the presence of SDS.

Figure 11a presents the effect of SDS, which is an ionic surfactant, on the NBs’ size. The NBs’ size decreased in the samples containing SDS compared to the samples with no additives present. However, bubble size remained approximately constant upon adding more than 3 times the critical micelle concentration (CMC = 8.2 × 10^–3^ M) of the surfactant. The CMC demonstrates the concentration that micelles start to be generated by surfactants^63^. The typical size of surfactant micelles such as SDS has been reported around 1.75 nm by other researchers, which is too small and challenging to be detected by NTA^50^. In our work, increasing SDS concentration to > 3 times the CMC resulted in micelle formation from the excess SDS amount rather than an increased interaction of SDS with NBs. This observation is consistent with Cho et al.^64^ who reported that the surface tension of NBs was decreased from 72 to 38 mN/m upon adding 5 times the CMC of SDS. In fact, SDS molecules provide a protective layer preventing nanobubbles from dissolving due to the adsorption of sulfate ions (R-SO_4_^−^) on the bubble interfaces^5^. Besides, in the presence of surfactant layers, the vaporization of volatile components is limited. Consequently, bubbles are stabilized by surfactants. Further, Fig. 11b indicates that the addition of SDS leads to an increase in the absolute value of zeta potential of the NBs. These results prove the adsorption of anions on the surface of NBs and the improvement of their electrostatic stabilization.

In other industrial techniques such as flotation, it seems that bubbles stabilized even before CMC of added surfactants. Inorganic salts are commonly used in flotation; the presence of inorganic salts in solution can have a significant impact on the adsorption behavior of surfactants at liquid–gas interfaces^65^. This phenomenon occurs due to the influence of counter ions, which are ions of opposite charge to the surfactant molecules. The interaction between counter ions and surfactants can increase their adsorption rate. In addition, salts can lower the CMC of surfactants by screening the electrostatic repulsions that occur between the head groups of the surfactant molecules^65^. Also, salts facilitate the migration of surfactant molecules towards the air–liquid interface^65^. Accordingly, the utilization of electrolytes alongside surfactants can lower the required surfactant concentration^65^. As a result, the presence of salts in the flotation process might be a contributing factor to the early stabilization of bubbles before reaching the CMC of added surfactants. The critical coalescence concentration (CCC) is another essential parameter for assessing surfactants and signifies the minimal concentration of a surfactant (i.e., frother) needed to avoid bubble coalescence during flotation^66^. Zhang et al.^67^ have observed that low bulk surfactant concentrations (below the CCC, which was lower than the CMC) have the most significant impact on bubble size reduction. This phenomenon can be explained by partitioning: the surfactant accumulates on the bubble surface, creating a "dry" froth with minimal water entrainment^67^. Partitioning, in this context, refers to the increased concentration of the surfactant in the froth compared to the bulk solution^67^. This concentration gradient of the surfactant (C_Interface_/C_Bulk_) between the bulk solution and the bubble interface acts as the primary driving force for reducing bubble size. Notably, the region with the most significant concentration gradient (C_Interface_/C_Bulk_) occurs at the lowest surfactant addition and exerts the most substantial influence in this regard^67^. With the addition of higher concentration of surfactants, C_Interface_/C_Bulk_ decreases until it reaches a constant value, at which point the bubble's size stabilizes. Indeed, the effectiveness of anti-coalescence agents, such as surfactants, depends on concentration and there is no further reduction in bubble size beyond CCC^68–70^. The maximum gradients in surface tension that can develop on the bubble surface are directly proportional to this concentration gradient (C_Interface_/C_Bulk_)^67^.

In this study, the high concentration of NBs offers a high gas–liquid interface, which presents more attractive regions for surfactant adsorption and provides more sites for interactions with surfactants. This might result in significantly increased C_Interface_/C_Bulk_ and high surface tension gradients, thus necessitating additional surfactant (3 × CMC) to stabilize bubbles. As the surfactant concentration approaches and surpasses the CMC, surfactant molecules start to self-assemble into micelles. During this process, the hydrophobic tails of surfactant molecules aggregate towards the core of the micelles, while the hydrophilic heads remain exposed to the surrounding liquid^71^. This self-assembly process results in a reduction in surface tension, which is likely to contribute to the decrease of the surface tension gradients on the bubbles’ surface, ultimately aiding in stabilizing the bubbles. Certainly, the complex interplay between surfactants, salts, and other factors in flotation requires further investigation to fully understand their combined impact on bubble stabilization. Therefore, understanding the mystery of the different types of surfactant effects on bubble motion and gas holdup in flotation columns represents a compelling area for further research.

### Effect of temperature on NBs

**Figure 12:**
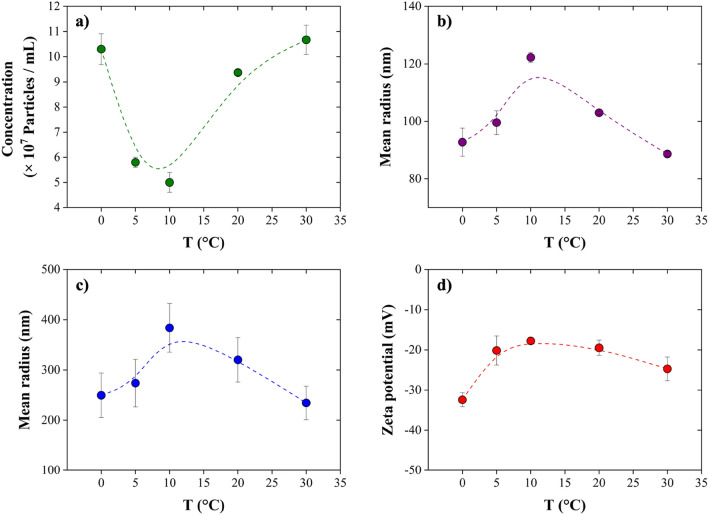
Impact of temperature on the (**a**) concentration and (**b**) mean radius of NBs obtained by NTA, (**c**) NBs mean radius obtained by DLS, and (**d**) zeta potential of NBs. As the temperature increases from 10 to 30 °C, the absolute value of zeta potential increases, which leads to higher concentrations of NBs with lower size in solution. Also, lowering the temperature from 10 to 0 °C causes an increase in the absolute value of zeta potential of NBs. Therefore, high concentration of NBs can be stable, as they shrink at lower temperature.

To study the effect of temperature on the NBs, the concentration, size, and zeta potential of NBs were recorded as a function of temperature. The findings unequivocally show that NBs are highly sensitive to temperature changes. Figure 12 demonstrates the characterization of bubbles over a thermal cycle between 0 and 30 °C. Accordingly, the concentration of NBs decreased from 10.3 ± 0.61 (× 10^7^ bubbles/mL) to 5 ± 0.4 (× 10^7^ bubbles/mL) upon increasing the storage temperature from 0 to 10 °C (Fig. 12a). However, NBs concentration increased up to 10.67 ± 0.58 (× 10^7^ bubbles/mL) upon heating the solution to 30 °C. Further, based on NTA data, the size of the bubbles increased from a radius of 92.75 ± 4.87 nm to 122.23 ± 1.72 nm as the storage temperature increased from 0 to 10 °C (Fig. 12b). However, their size decreased to 88.65 ± 0.92 nm upon a further increase of the storage temperature from 10 to 30 °C. Accordingly, a temperature of 10 °C could be considered as a transition point for the behavior of NBs, a trend which was also confirmed by DLS measurements (Fig. 12c). A similar trend has been reported by Li et al.^47^ for the temperature range between 10 and 30 °C, as they observed a decrease of around 20% in the size of NBs upon heating a solution containing air NBs from 10 to 30 °C.

Zeta potential measurements at different temperatures indicated that there is no monotonic relationship between zeta potential and temperature (Fig. 12d)^47^. As the temperature increased from 0 to 10 °C, the absolute value of zeta potential decreased before increasing in the temperature range between 10 and 30 °C. The effect of temperature on zeta potential is not well described in literature. A complex interaction between temperature and zeta potential occurs as a result of alterations to ionic distributions, adsorption equilibria, and charging characteristics^47,72,73^. To interpret the effect of temperature on NBs, two different mechanisms should be considered. First, the self-ionization of water, which releases OH^−^ ions in aqueous solutions. Due to the endothermic nature of this reaction, increasing the temperature increases the concentration of OH^−^. Also, in an inert hydrophobic interface, macroscopic measurements and theoretical predictions have established that water tends to form negatively charged surfaces at neutral pH through the preferential adsorption of hydroxide ions (OH^−^)^47,74^. The negativity of the zeta potential of NBs is supported by this theory. Therefore, the excess OH^−^ formed when increasing the solution temperature gets adsorbed on the bubble surfaces, thus increasing the negative charge and the stability of NBs. However, this increase in the bubbles’ charge is offset by a second mechanism, namely the temperature-dependent mobility of ions. The mobility of ions is enhanced by increasing temperature. By increasing ion mobility, OH^−^ ions are less likely to adsorb to bubble surfaces^47^, while higher ion mobility also tends to increase the chance of bubble coalescence. Therefore, in this point of view, a temperature increase tends to decrease the stability of NBs.

**Figure 13:**
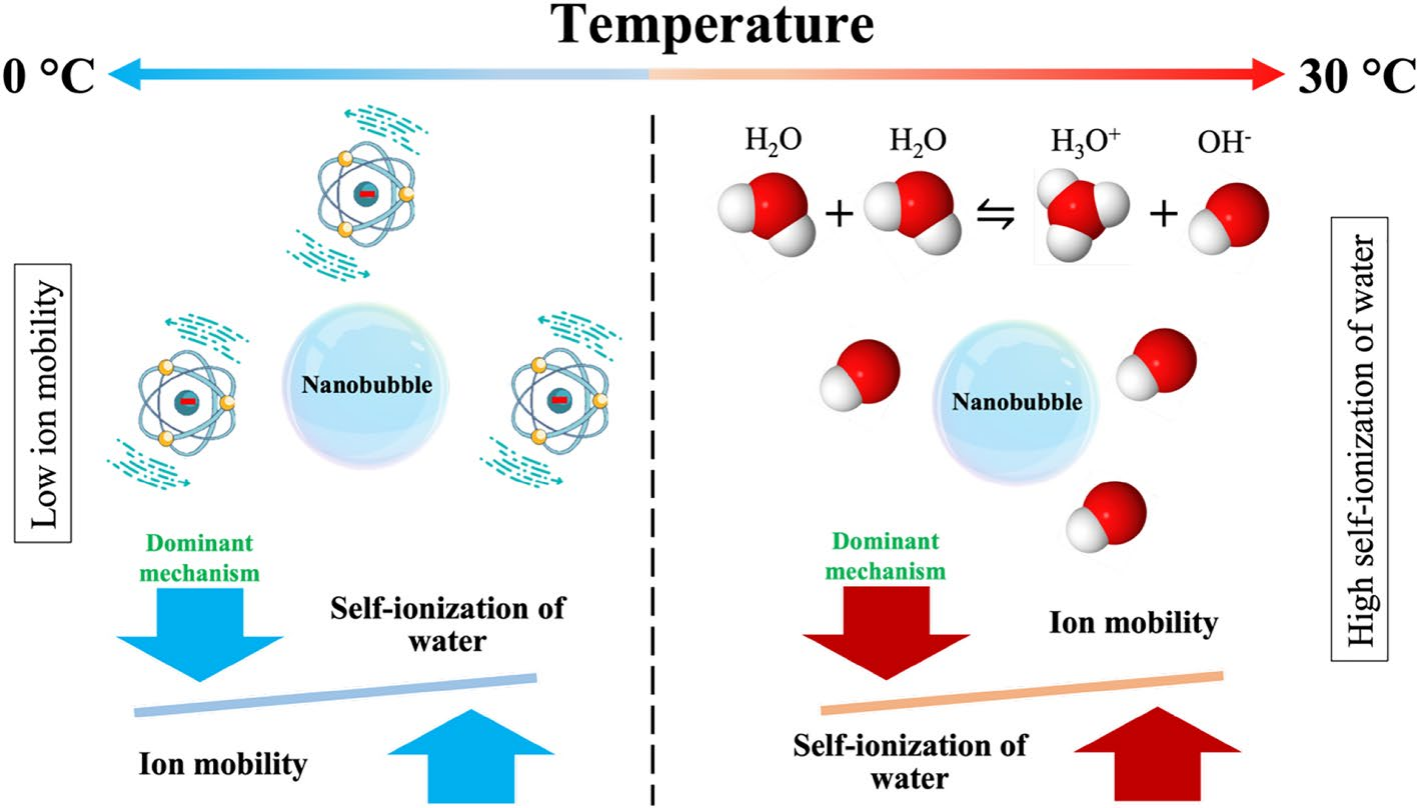
There are two competing mechanisms, based on the self-ionization of water and ion mobility, that explain the effect of temperature on NBs. Ion mobility is dominant at low temperatures (from 10 to 0 °C) and suggests that the mobility of ions is decreased at low temperature, reducing bubble coalescence and agglomeration. On the contrary, self-ionization of water controls the stability of NBs at high temperature (between 10 and 30 °C), suggesting that more OH^−^ ions are released to the bulk solution and adsorbed onto the NBs surfaces, which in turn stabilize the NBs.

A summary of these two competing mechanisms of self-ionization of water and ion mobility determines the effect of temperature on NBs, as shown in Fig. 13. When the temperature increases from 10 to 30 °C, the self-ionization of water theory is dominant; consequently, more OH^−^ ions are released to the bulk solution, which in turn favors their adsorption to NBs surfaces. As a result, the absolute value of zeta potential increases, which leads to higher concentrations of stable NBs with lower size in solution. However, the ion mobility mechanism overcomes and controls the system in the temperature range of between 0 and 10 °C. Accordingly, the mobility of ions, which leads to a desorption of OH^−^ from the NBs surfaces, decreases when lowering the temperature from 10 to 0 °C. Hence, the absolute value of zeta potential of NBs increases, while the coalescence and agglomeration of bubbles are reduced. Therefore, a high concentration of stable NBs can be expected at lower temperature.

In addition, there are other factors, namely surface tension and gas solubility, that should be taken into account when interpreting the effect of temperature on NBs. Variations in water temperatures can influence the surface tension of water, which, in turn, has the potential to indirectly alter the stability of NBs. Surface tension decreases with increasing temperature from 10 to 30 °C, which could cause a reduction in NBs size according to the Young–Laplace equation^29^. Moreover, the size of NBs was found to vary with temperature due to the impact of temperature on gas solubility^29,44^. Gas molecules diffuse from regions of higher concentration (inside the NBs) to regions of lower concentration (in the liquid). If the solubility of the gas is high, a significant number of gas molecules can dissolve into the liquid, resulting in a reduction in the size of the NBs^44^. Accordingly, lowering the temperature, from 10 to 0 °C, leads to an increase of gas solubility, which in turn leads to a decrease of the NBs size.

## Conclusions

The characteristics of air nanobubbles (NBs), generated through hydrodynamic cavitation, were investigated. Air NBs were produced in deionized water at a concentration of 1.2 × 10^8^ bubbles/mL and a mean radius of 84.66 ± 7.88 nm. Their stability, concentration, size, and zeta potential were investigated by DLS and NTA for two months. The concentration of NBs was found to decrease over time, whereas their size increased. Nevertheless, around 26% of stable NBs reported in solution after two months. Additionally, the effect of electrolytes (dissolved NaCl and Na_2_SO_4_) on the stability of air NBs was studied in a widespread concentration range. A small amount of NaCl and Na_2_SO_4_ salt decreased the size of the NBs; however, higher salt concentrations, up to 1 M, caused a significant increase in the NBs size. At the same salt concentrations, 1.97 up to 17.29% larger NBs were detected in the sulfate solutions compared to chloride ones, thus proving a significant effect of the electrolyte anion on the NBs stability. Further, by adding SDS, the negativity of zeta potential of NBs was increased from − 19.48 ± 1.89 to − 68.82 ± 3.46 mV, which proves that the presence of surfactants in solution leads to higher NBs stability. Accordingly, the size of NBs was reduced upon the addition of SDS. Furthermore, a set of pH experiments indicated that alkaline solutions are more favorable for the stability of NBs compared to acidic ones. The zeta potential of NBs was almost zero at pH 4 for both HCl and H_2_SO_4_ solutions; though, the absolute value of NBs’ zeta potential was increased to − 23.84 ± 1.74 mV at pH 9. Finally, an extensive experimental study is presented to probe the response of bulk NBs to a range of environmental temperatures, namely from 0 to 30 °C. The results showed that a high concentration of small NBs is stable at 0 °C. However, the number of NBs decreases, and their size increases by increasing the temperature up to 10 °C. Then again, the concentration of NBs increases, while their size decreases, upon increasing the temperature further to 30 °C. The effect of temperature on NBs stability can be interpreted by considering two competing mechanisms, namely the self-ionization of water and the ion mobility. Overall, the results presented in this work are essential for the development of innovative environmental applications for the treatment of mining effluents, based on NBs technology.

## Data Availability

All data generated or analyzed during this study are included in this published article.
